# Prevalence Estimates of Gestational Diabetes Mellitus in the United States, Pregnancy Risk Assessment Monitoring System (PRAMS), 2007–2010

**DOI:** 10.5888/pcd11.130415

**Published:** 2014-06-19

**Authors:** Carla L. DeSisto, Shin Y. Kim, Andrea J. Sharma

**Affiliations:** **Author Affiliations:** Shin Y. Kim, Andrea J. Sharma, Centers for Disease Control and Prevention, Atlanta, Georgia.

## Abstract

**Introduction:**

The true prevalence of gestational diabetes mellitus (GDM) is unknown. The objective of this study was 1) to provide the most current GDM prevalence reported on the birth certificate and the Pregnancy Risk Assessment Monitoring System (PRAMS) questionnaire and 2) to compare GDM prevalence from PRAMS across 2007–2008 and 2009–2010.

**Methods:**

We examined 2010 GDM prevalence reported on birth certificate or PRAMS questionnaire and concordance between the sources. We included 16 states that adopted the 2003 revised birth certificate. We also examined trends from 2007 through 2010 and included 21 states that participated in PRAMS for all 4 years. We combined GDM prevalence across 2-year intervals and conducted *t* tests to examine differences. Data were weighted to represent all women delivering live births in each state.

**Results:**

GDM prevalence in 2010 was 4.6% as reported on the birth certificate, 8.7% as reported on the PRAMS questionnaire, and 9.2% as reported on either the birth certificate or questionnaire. The agreement between sources was 94.1% (percent positive agreement = 3.7%, percent negative agreement = 90.4%). There was no significant difference in GDM prevalence between 2007–2008 (8.1%) and 2009–2010 (8.5%, *P* = .15).

**Conclusion:**

Our results indicate that GDM prevalence is as high as 9.2% and is more likely to be reported on the PRAMS questionnaire than the birth certificate. We found no statistical difference in GDM prevalence between the 2 phases. Further studies are needed to understand discrepancies in reporting GDM by data source.

## Introduction

Gestational diabetes mellitus (GDM) is defined as impaired glucose tolerance with onset or first recognition during pregnancy (1). Women with GDM are at high risk for pregnancy and delivery complications including infant macrosomia, neonatal hypoglycemia, and cesarean delivery (2,3). Additionally, women who are affected by GDM have more than a 7-fold increased risk of developing type 2 diabetes 5 to 10 years after delivery (4). Children born to mothers with GDM are also more likely to develop impaired glucose tolerance (5). Risk factors for GDM include higher parity, advanced maternal age, family history of diabetes mellitus, nonwhite race, and overweight and obesity (6–8).

Although the true prevalence of GDM is unknown, GDM is estimated to affect 1% to 14% of pregnancies in the United States annually, depending on the population studied and the diagnostic tests used (9–11). GDM prevalence has been steadily increasing with the rise of obesity and type 2 diabetes (12). Both birth certificates and the Pregnancy Risk Assessment Monitoring System (PRAMS), which includes a questionnaire completed by mothers, can provide population-based prevalence estimates of GDM. However, birth certificate data are limited in that in 2010, only 33 states and the District of Columbia, representing 76% of all 2010 births in the United States, had implemented the 2003 revised birth certificate, which differentiates pre-pregnancy diabetes from GDM (13). Moreover, studies indicate that whereas specificity for GDM is high on the birth certificate, sensitivity is as low as 48% (14). Thus, GDM prevalence obtained from the birth certificate alone is likely underestimated. In contrast, PRAMS may overestimate GDM prevalence. CDC periodically updates its PRAMS questions to reflect evidence-based revisions, with each revised version constituting a new phase. In a validation study of the PRAMS phase 5 (2007–2008) GDM question across 5 states, 61.8% of 277 study participants who reported GDM on the questionnaire did not have a GDM diagnosis in their prenatal or hospital medical records (15). In an attempt to reduce misclassification of GDM diagnosis based on maternal self-report, PRAMS revised the GDM question for phase 6 (15,16). On the basis of this PRAMS phase 5 validation study, which found that the PRAMS question substantially overestimated the GDM prevalence, we hypothesized that the change in the GDM question would result in a lower prevalence of GDM.

The objective of this analysis was 1) to provide a current estimate of GDM prevalence among PRAMS states using the most recent data available (2010) and 2) to compare GDM prevalence from the phase 5 (2007–2008) and phase 6 (2009–2010) PRAMS questionnaires.

## Methods

### Study population

We analyzed 2007–2010 data from PRAMS, an ongoing, state-based, population-based surveillance system that collects information about maternal behaviors before, during, and after pregnancies that result in live births. Using birth certificates, PRAMS researchers sample live births delivered within the previous 2 to 4 months. Self-administered questionnaires are mailed to mothers’ homes; nonresponders are followed up by telephone. Each questionnaire is linked to the respondent’s child’s birth certificate. More details on PRAMS are available at www.cdc.gov/prams.

GDM was ascertained from both the birth certificate and the PRAMS questionnaire. We defined GDM as present if it was reported on either data source and the woman did not report a diagnosis of prepregnancy diabetes on the questionnaire. The birth certificate variable is based on a check box that differentiates prepregnancy diabetes (diagnosis before this pregnancy) from gestational diabetes (diagnosis in this pregnancy). During PRAMS phase 5 (2005–2008), women were asked to select from a list any problems they had during their most recent pregnancy, including “high blood sugar (diabetes) that started during this pregnancy.” Beginning in 2009 (PRAMS phase 6), women were asked, “During your most recent pregnancy, were you told by a doctor, nurse, or other health care worker that you had gestational diabetes (diabetes that started during this pregnancy)?” Women who responded yes to either question were considered to have GDM for the respective survey.

For both PRAMS phase 5 and phase 6, we obtained data from the birth certificate on maternal race/ethnicity; education; marital status; parity; enrollment in the Special Supplemental Nutrition Program for Women, Infants, and Children (WIC); and Medicaid. Maternal race/ethnicity was characterized as non-Hispanic white, non-Hispanic black, Hispanic, American Indian/Alaskan Native, Asian/Pacific Islander, and other. On the 2003 birth certificate, respondents were able to select Hispanic ethnicity and a separate race category. We categorized anyone who reported Hispanic ethnicity as Hispanic, regardless of any secondary race classification. We categorized Chinese, Japanese, Filipino, Hawaiian, and “Other Asian” as Asian/Pacific Islander. “Other” includes those who reported mixed race or any race/ethnicity other than those described above. The state of Vermont released their race/ethnicity data only as non-Hispanic white or “other.”

Maternal education was characterized as less than 12 years, 12 years, or 13 or more years, depending on the highest level of school completed. Marital status was characterized as married (at birth, conception, or any time between) or other. Parity was characterized as 0, 1, or 2 or more. We used Medicaid and WIC enrollment as proxy indicators of socioeconomic status.

### Statistical analysis

We examined demographic factors, including maternal age, race/ethnicity, parity, maternal education, marital status, WIC enrollment, and Medicaid, overall and by GDM prevalence for 2010. We used χ^2^ tests to examine differences in GDM prevalence by demographics. We then calculated the 2010 prevalence overall and by geographic area according to reporting sources. Furthermore, we examined concordance between the 2 sources by calculating the percent agreement.

We combined estimated GDM prevalence across 2-year intervals in PRAMS phase 5 (2007–2008) and phase 6 (2009–2010) and then conducted *t *tests to examine whether there were differences in the prevalence of GDM as reported by the different questionnaires.

States were included in our GDM prevalence estimates if they had adopted the 2003 revised birth certificate and met the 65% response rate threshold for the PRAMS questionnaire in 2010. Fifteen states and New York City met these criteria. Twenty-one states were included in our comparison of phase 5 and phase 6 PRAMS questionnaires because they met the 65% response threshold for the PRAMS questionnaire for 2007–2010.

PRAMS data are weighted to represent all women delivering live births within each state, adjusting for sampling design, noncoverage, and nonresponse. We used SAS-callable SUDAAN 10.0.1 (Research Triangle Park, North Carolina) for all statistical analyses. We assessed significance at *P* < .05.

## Results

### 2010 Prevalence estimates of GDM

Records from 23,479 women in 2010, representing 1,353,810 births, were included in our analysis. In 2010, PRAMS survey participants were predominantly non-Hispanic white, having their first child, married, had more than a high school education, and were not enrolled in WIC or Medicaid (Table 1). GDM prevalence increased with maternal age, number of children, and WIC use, and decreased with higher education. Prevalence varied by maternal race/ethnicity; Asian/Pacific Islanders and Hispanics had the highest prevalence estimates (16.3% and 12.1%, respectively). Non-Hispanic whites had the lowest prevalence estimate (6.8%). GDM prevalence by maternal characteristic using the birth certificate only and the PRAMS questionnaire only showed similar results.

**Table 1:** Maternal Characteristics and GDM Prevalence For 15 States and New York City, Pregnancy Risk Assessment Monitoring System (PRAMS) 2010^a^

Characteristic	Sample with Maternal Characteristic, % (SE)	GDM by Maternal Characteristic, % (SE)^b^
**Maternal age, y^c^**		
<20	9.6 (0.4)	6.0 (1.1)
20–24	23.0 (0.5)	5.9 (0.6)
25–29	28.8 (0.5)	8.2 (0.6)
30–34	24.3 (0.5)	11.1 (0.8)
≥35	14.3 (0.4)	15.5 (1.2)
**Maternal race/ethnicity^c^**		
Non-Hispanic white	52.8 (0.4)	6.8 (0.4)
Non-Hispanic black	13.9 (0.3)	10.5 (0.9)
Hispanic	25.9 (0.3)	12.1 (0.9)
American Indian/Alaska Native	0.7 (0.1)	8.9 (2.1)
Asian/Pacific Islander	4.3 (0.2)	16.3 (1.8)
Other	2.5 (0.2)	10.0 (2.4)
**Parity^c^**		
0	40.5 (0.6)	7.8 (0.5)
1	31.0 (0.6)	9.0 (0.7)
≥2	28.5 (0.6)	11.4 (0.8)
**Maternal education, y^c^**		
<12	20.0 (0.5)	12.3 (1.0)
12	25.0 (0.5)	9.4 (0.8)
≥13	55.0 (0.6)	7.9 (0.4)
**Marital status**		
Married	60.2 (0.6)	9.1 (0.4)
Other	39.8 (0.6)	9.3 (0.6)
**WIC enrolled^c^**		
Yes	49.2 (0.6)	10.8 (0.6)
No	50.8 (0.6)	7.6 (0.4)
**Health insurance**		
Medicaid	43.2 (0.6)	9.9 (0.5)
Other	56.8 (0.6)	8.7 (0.5)

Abbreviations: GDM, gestational diabetes mellitus; SE, standard error; WIC, Special Supplemental Nutrition Program for Women, Infants, and Children.

a States and metropolitan areas included were Colorado, Delaware, Georgia, Maryland, Missouri, Nebraska, New York, New York City, Ohio, Oklahoma, Oregon, Texas, Utah, Vermont, Washington, and Wyoming.

b GDM prevalence estimates were calculated for women who had GDM reported either on the birth certificate or PRAMS questionnaire but did not self-report pre-pregnancy diabetes.

c Difference in prevalence of GDM by maternal characteristic (*P* < .05) by χ^2^ test.

The prevalence of GDM was 9.2% in 2010 among the 15 states and New York City when either the birth certificate or the PRAMS questionnaire indicated a GDM diagnosis (Table 2). The prevalence from the birth certificate alone was 4.6% and the prevalence from the PRAMS questionnaire alone was 8.7%. The percent agreement between the birth certificate and questionnaire was 94.1%. The percent positive agreement was 3.7% and the percent negative agreement was 90.4%. Overall, 5% of mothers who self-reported GDM diagnosis on the questionnaire did not have GDM recorded on the birth certificate and 0.9% of mothers who had GDM reported on their birth certificate did not self-report a GDM diagnosis on the questionnaire. Across both data sources, Wyoming had the lowest GDM prevalence (range, 2.2%–5.5%) and New York City had the highest prevalence (range, 5.4%–11.7%) (Table 2).

**Table 2:** Comparison of GDM Prevalence by Data Source, 15 States and New York City, Pregnancy Risk Assessment Monitoring System (PRAMS) 2010

State or Metropolitan Area	BC Only, % (SE)	PRAMS Questionnaire Only, % (SE)	Both BC and PRAMS Questionnaire, % (SE)	Either BC or PRAMS Questionnaire, % (SE)
Total	4.6 (0.3)	8.7 (0.3)	3.7 (0.2)	9.2 (0.4)
Colorado	4.7 (0.7)	6.1 (0.8)	3.7 (0.6)	7.1 (0.8)
Delaware	3.5 (0.6)	8.3 (0.8)	3.1 (0.5)	8.3 (0.8)
Georgia	4.6 (1.1)	7.8 (1.3)	3.2 (0.9)	8.9 (1.4)
Maryland	3.9 (0.7)	8.5 (1.1)	3.2 (0.6)	8.9 (1.1)
Missouri	5.3 (0.7)	7.0 (0.8)	3.7 (0.5)	8.2 (0.8)
Nebraska	4.4 (0.6)	9.4 (0.8)	3.5 (0.5)	9.9 (0.8)
New York	4.3 (0.8)	6.5 (1.0)	3.3 (0.7)	6.7 (1.0)
New York City	6.2 (0.8)	11.5 (1.1)	5.4 (0.8)	11.7 (1.1)
Ohio	4.8 (0.8)	8.3 (1.0)	4.2 (0.7)	9.0 (1.1)
Oklahoma	3.1 (0.7)	8.2 (1.0)	2.0 (0.5)	8.6 (1.1)
Oregon	6.4 (0.8)	10.4 (1.0)	5.1 (0.7)	11.0 (1.0)
Texas	4.0 (0.6)	10.0 (0.9)	3.3 (0.6)	10.3 (0.9)
Utah	4.0 (0.5)	6.4 (0.7)	3.5 (0.5)	6.6 (0.7)
Vermont	4.2 (0.6)	6.9 (0.8)	3.9 (0.6)	7.0 (0.8)
Washington	5.0 (0.6)	8.8 (0.8)	4.0 (0.6)	9.3 (0.9)
Wyoming	3.0 (0.6)	4.8 (0.7)	2.2 (0.5)	5.5 (0.7)

Abbreviations: GDM, gestational diabetes mellitus; BC, birth certificate; SE, standard error.

### GDM estimates from PRAMS phase 5 and phase 6

Records from 123,373 women from 2007–2010, representing 4,223,575 births, were included in our analysis. In the 21 states that participated in both phases, there was no significant difference between the GDM prevalence from the phase 5 PRAMS questionnaire (8.1%) and the prevalence from the phase 6 questionnaire (8.5%) (*P* = .15) (Table 3). There was also no significant difference between the GDM prevalence from PRAMS phase 5 and phase 6 in any of the 21 states. In both phases, Utah had the lowest prevalence (5.7% in phase 5 and 5.6% in phase 6) and Rhode Island had the highest prevalence (10.4% in phase 5 and 11.7% in phase 6).

**Table 3:** Prevalence of GDM by State From Pregnancy Risk Assessment Monitoring System (PRAMS) Questionnaires, 2007–2010

State or Metropolitan Area	GDM Prevalence from PRAMS Phase 5, 2007–2008, % (SE)^a^	GDM Prevalence from PRAMS Phase 6, 2009–2010, % (SE)^b^	*P* Value^c^
Total	8.1 (0.2)	8.5 (0.2)	.15
Alaska	7.8 (0.7)	7.7 (0.7)	.93
Arkansas	9.2 (0.7)	10.0 (0.8)	.46
Colorado	5.9 (0.5)	6.8 (0.6)	.22
Delaware	8.1 (0.6)	7.7 (0.6)	.69
Georgia	7.6 (1.0)	8.8 (1.0)	.42
Hawaii	10.2 (0.5)	11.3 (0.7)	.24
Maine	9.2 (0.7)	9.4 (0.7)	.83
Maryland	8.4 (0.7)	8.9 (0.8)	.59
Massachusetts	6.9 (0.6)	6.5 (0.6)	.62
Minnesota	7.7 (0.5)	7.9 (0.6)	.80
Nebraska	8.1 (0.6)	8.9 (0.6)	.28
New Jersey	9.9 (0.6)	9.1 (0.6)	.31
Ohio	8.3 (0.7)	9.3 (0.8)	.36
Oklahoma	9.2 (0.8)	7.8 (0.7)	.18
Oregon	9.2 (0.8)	10.1 (0.7)	.39
Rhode Island	10.4 (0.7)	11.7 (0.8)	.22
Utah	5.7 (0.4)	5.6 (0.4)	.98
Vermont	6.1 (0.5)	6.5 (0.5)	.60
Washington	8.2 (0.6)	9.2 (0.6)	.24
West Virginia	9.0 (0.7)	10.1 (0.7)	.25
Wyoming	6.0 (0.6)	5.6 (0.6)	.63

a Questionnaire data are based on respondents being asked to select from a list any problems they had during their most recent pregnancy, including “high blood sugar (diabetes) that started during this pregnancy.”

b Questionnaire data are based on self-reported answers to the question “During your most recent pregnancy, were you told by a doctor, nurse, or other health care worker that you had gestational diabetes (diabetes that started during this pregnancy)?”

c Calculated by *t* test.

## Discussion

Our data suggests that the prevalence of GDM in 2010 was between 4.6% (as reported on the birth certificate only) and 9.2% (as reported on either the birth certificate or PRAMS questionnaire). Although there was high agreement (94.1%) between the 2 sources, more cases of GDM are identified through the PRAMS questionnaire than from the birth certificate. In our analysis, 5% of mothers who self-reported GDM diagnosis on the questionnaire in 2010 did not have GDM recorded on the birth certificate. Birth certificate data have been shown to underestimate GDM prevalence. In a comprehensive literature review summarizing the validity of birth certificate data for identifying diabetes-complicated births, the sensitivity for identifying GDM ranged from 46% to 83%, with a median of 65%; specificity was consistently above 98% (14).

In PRAMS phase 5, women were asked if they had high blood glucose during this pregnancy. Because GDM diagnosis is based on a 2-step test where women who have positive results on the first glucose challenge test go on to an oral glucose tolerance test, only women who have positive results on both tests would be diagnosed with GDM. Some women who had positive results on the first test but not the second may respond that they had high blood glucose in this pregnancy. In PRAMS phase 6, the wording of the question was changed so that mothers were asked if they were told by a clinician they specifically had “gestational diabetes” instead of “high blood sugar.” We hypothesized that this wording change would reduce misclassification and result in a lower GDM prevalence in phase 6 compared with phase 5. However, we found no significant difference in GDM prevalence between the 2 phases. We do not know if this is evidence that the phase 6 question continues to overestimate true GDM prevalence or if it reflects a true increase in GDM prevalence. However, a recent study of 100 women in Utah who reported GDM on the phase 6 questionnaire but had no indication of GDM on their child’s birth certificate reported that 42% of these women did not have a GDM diagnosis in their medical records (15). Therefore, given the current limitations of both the birth certificate and PRAMS, true GDM prevalence is likely between the estimates obtained from the 2 sources.

An additional challenge in determining GDM prevalence is that prevalence is very sensitive to the diagnostic criteria used. In recent years criteria have changed and there has been a lack of consensus about which criteria to use (17). The 3 primary criteria used in the United States are by the National Diabetes Data Group (18), Carpenter and Coustan (19), and the International Association of Diabetes and Pregnancy Study Groups (IADPSG) (20). The first 2 criteria are recommended by the American Congress of Obstetricians and Gynecologists (ACOG) and result in a GDM diagnosis in about 5% to 6% of pregnancies, whereas the latter is recommended by the American Diabetes Association (1) and results in a GDM diagnosis in about 18% to 20% of pregnancies (21). Recently, the National Institutes of Health Consensus Development Conference concluded that “there is not sufficient evidence to adopt . . .the IADPSG [diagnostic criteria]” (21). During our study period it is likely that a mix of these criteria was used, but neither the birth certificate nor PRAMS ascertains which criteria were used for GDM diagnoses. Further studies are needed to identify which criteria are most commonly used by physicians.

Even if our most conservative estimate is used, GDM still affects nearly 1 in 20 (4.6%) pregnancies in the United States, meaning a substantial number of women are at greater risk for obstetric complications (2) and for developing type 2 diabetes later in life (4). The children born to these mothers are also more likely to develop impaired glucose tolerance and metabolic complications (22). In addition, Chen and colleagues found that GDM increased US medical costs by $636 million in 2007 (10). Moreover, approximately 36% of GDM-related medical costs are covered by government programs, primarily Medicaid (10).

A recent study showed that GDM rates differ by state, with the greatest variation attributable to differences in obesity (23). Obesity is known to be associated with GDM (24); nearly 50% of GDM could potentially be prevented if we reduced the risk of overweight and obesity to that of normal-weight women (25). Preventing obesity is a key component of well woman care, regardless of pregnancy intentions. Since half of US pregnancies are unintended (26), maintaining a healthy weight throughout the reproductive years benefits women and improves the health of any future pregnancies. Emphasis should be placed on ensuring access to weight management counseling and treatment as a standard component of routine care, particularly among high-risk groups. Both the Centers for Disease Control and Prevention (CDC) and ACOG recommend well woman care, including obesity screening (27,28). Counseling about nutrition and physical activity, as well as appropriate contraceptive use, may help women achieve a healthy weight before pregnancy. However, lack of providers offering this kind of care, public awareness to seek well woman services, and insurance coverage of those services represent significant barriers to access (28,29).

This analysis includes a few limitations. Our 2010 GDM prevalence estimate is limited to those states that adopted the 2003 revised birth certificate and provided PRAMS data. The 15 states and New York City included in our analysis represent approximately one-third of births in 2010 and therefore may not be representative of the entire United States. Additionally, the respondents included in this study may differ from those who were excluded. PRAMS excludes women who had stillbirths or fetal deaths, which may be associated with gestational diabetes (30). Finally, our estimates are based on administrative and self-report data and are therefore subject to reporting biases.

The true prevalence of GDM is unknown, and estimates rely on data sources that all have limitations. Our analysis adds 2 additional data points to the literature for population-based prevalence estimates of GDM for 2010 and also shows how the data sources — the birth certificate and mothers’ self-report on the PRAMS questionnaire — relate to one another. The true prevalence of GDM likely lies between our prevalence estimates of 4.6% and 9.2%. Our results indicate that GDM prevalence is high in the United States, and we found no evidence of change between 2007 and 2010. However, there is a need for understanding the discrepancies in GDM prevalence estimates by data sources and for a consensus on which diagnostic criteria to use. GDM is a significant public health concern because of its long-term implications for maternal and child health. This study provides additional information about prevalence of GDM, which is important for understanding the future burden of related diseases, most notably type 2 diabetes, and related health care costs.
